# Efficacy of inhaled nitric oxide in preterm infants ≤ 34 weeks: a systematic review and meta—analysis of randomized controlled trials

**DOI:** 10.3389/fphar.2023.1268795

**Published:** 2024-01-11

**Authors:** Zhoushan Feng, Xiaohong Wu, Xiaona Xu, Qiliang Cui, Fan Wu

**Affiliations:** ^1^ Department of Neonatology, Guangzhou Key Laboratory of Neonatal Intestinal Diseases, The Third Affiliated Hospital of Guangzhou Medical University, Guangzhou, China; ^2^ Department of Obstetrics and Gynecology, Guangdong Provincial Key Laboratory of Major Obstetric Diseases, Guangdong Provincial Clinical Research Center for Obstetrics and Gynecology, Guangdong-Hong Kong-Macao Greater Bay Area Higher Education Joint Laboratory of Maternal-Fetal Medicine, Guangzhou, China; ^3^ Department of Obstetrics and Gynecology, School of Medicine, The International Peace Maternity and Child Health Hospital, Shanghai Jiao Tong University, Shanghai, China

**Keywords:** nitric oxide, bronchopulmonary dysplasia, mortality, premature infant, meta analysis

## Abstract

**Background:** The effect of inhaled nitric oxide (iNO) in neonates >34 weeks on improving respiration is well documented. However, the efficacy of iNO in preterm infants ≤34 weeks remains controversial.

**Objectives:** The main purpose of this review is to assess the effectiveness and safety of iNO treatment in preterm infants ≤34 weeks.

**Search methods:** We systematically searched PubMed, Embase and Cochrane Libraries from their inception to 1 June 2023. We also reviewed the reference lists of retrieved studies.

**Selection criteria:** Our study involved randomized controlled trials on preterm infants ≤34 weeks, especially those receiving iNO treatment, and mainly assessed outcomes such as bronchopulmonary dysplasia (BPD) and mortality. Two authors independently reviewed these trials, extracted data, and evaluated study biases. Disagreements were resolved by consensus. We used the GRADE method to assess evidence quality.

**Results:** Our research included a total of 17 studies involving 4,080 neonates and 7 follow-up studies. The synthesis of results showed that in neonates, iNO treatment reduced the incidence of BPD (RR: 0.92; 95% CI: 0.86–0.98). It also decreased the composite outcome of death or BPD (RR: 0.94; 95% CI: 0.90–0.98), without increasing the risk of short-term (such as intraventricular hemorrhage, periventricular leukomalacia) and long-term neurological outcomes (including Bayley mental developmental index <70, cerebral palsy and neurodevelopmental impairment). Furthermore, iNO did not significantly affect other neonatal complications like sepsis, pulmonary hemorrhage, necrotizing enterocolitis, and symptomatic patent ductus arteriosus. Subgroup analysis revealed that iNO significantly reduced BPD incidence in neonates at 36 weeks under specific intervention conditions, including age less than 3 days, birth weight over 1,000 g, iNO dose of 10 ppm or higher, or treatment duration exceeding 7 days (*p* < 0.05).

**Conclusion:** Inhaled NO reduced the incidence of BPD in neonates at 36 weeks of gestation, and the effect of the treatment depended on neonatal age, birth weight, duration and dose of iNO. Therefore, iNO can be considered a promising treatment for the potential prevention of BPD in premature infants. More data, however, would be needed to support nitric oxide registration in this specific patient population, to minimize its off-label use.

## Introduction

In recent decades, the survival rate of premature infants has increased significantly due to prenatal hormones and supplementation with pulmonary surfactant (McGoldrick et al., 2020; Wu et al., 2021). However, due to immature lung development, premature infants are prone to oxidative stress, an inflammatory attack and other complications, leading to damage to the physiological structure of the lung and impaired lung function (Speer, 2006; Cannavò et al., 2021). In the short term, acute respiratory failure diseases, such as respiratory distress syndrome and pulmonary hypertension, often occur (Wu et al., 2019; Mandell et al., 2021); however, bronchopulmonary dysplasia (BPD), a chronic lung disease, is currently the most common respiratory disease in preterm infants that prolongs hospitalization and predisposes them to neurological complications (Majnemer et al., 2000; Lin et al., 2017). Severe BPD is closely related to an inflammatory attack, pulmonary hypertension and an abnormal lung physiology due to an angiogenic disorder or alveolar fibrosis (Thebaud et al., 2019).

As a signaling molecule, NO is synthesized from L-arginine by NO synthase in various human cells (Forstermann and Sessa, 2012). In addition to selectively dilating pulmonary blood vessels and promoting lung maturation, NO can also inhibit the expression of proinflammatory factors and the release of inflammatory mediators in the lung (ter Horst et al., 2007; Krasuski et al., 2000; Rose et al., 2010). Therefore, theoretically, NO can effectively prevent or treat BPD. Clinically, NO has been shown to be a specific drug for improving short-term oxygenation in neonates and a treatment for persistent pulmonary hypertension (Goldman et al., 1996; Dani et al., 2017). A meta-analysis showed that iNO improved short-term oxygenation and decreased ECMO use in term or near-term infants (Barrington et al., 2017a).

However, the current clinical studies of NO for preventing or reducing the incidence of BPD in preterm infants are controversial. In a multicenter randomized controlled study, Hasan et al. reported that iNO did not confer a significant benefit on the composite outcome of BPD or mortality or on long-term neurological outcomes (Hasan et al., 2017). On the other hand, Schreiber et al. found that iNO reduced the incidence of the composite outcome of death or BPD and improved short-term or long-term neurological outcomes such as intraventricular hemorrhage (IVH), periventricular leukomalacia (PVL), or cerebral palsy (CP) (Schreiber et al., 2003). In contrast, in another multicenter study by Van Meurs et al., iNO not only failed to provide an overall benefit but also increased the incidence of mortality (Van Meurs et al., 2005). Therefore, our study systematically searched for randomized controlled trials (RCTs) involving the treatment of preterm infants ≤34 weeks of gestation with iNO and conducted a meta-analysis. This was undertaken to further elucidate the efficacy of iNO on the incidence of neonatal BPD and/or mortality, as well as its impact on short-term and long-term neurological outcomes, including conditions such as IVH, PVL and CP.

## Materials and methods

This systematic review was meticulously carried out in alignment with the Preferred Reporting Items for Systematic Reviews and Meta-Analyses (PRISMA) guidelines (Page et al., 2021), as detailed in the Supplementary Table S1 checklist. The protocol for this review was published on PROSPERO with the registration number CRD42022315208.

### Search strategy

Two reviewers (X.W. and Z.F.) conducted a systematic search of the literature to identify all RCTs focusing on the treatment of inhaled NO in premature infants ≤34 weeks of gestation. We searched major databases including MEDLINE *via* PubMed, EMBASE, and the Cochrane Library, covering records from their inception to 01 June 2023. This extensive search was strategically designed to encompass a wide array of potentially relevant studies, with the limitation that only English language papers were considered. A specific search strategy was tailored for each database to ensure the maximum relevance and breadth of our collected data. The specifics of these search terms and strategies, including any necessary adjustments made for the different databases, are detailed in Supplementary Table S2. In addition to this database search, we also conducted a manual review of the references listed in each included study. This step was crucial in identifying any pertinent papers that might have been overlooked during the initial search phase. However, we recognize, in line with AMSTAR 2 criteria (Shea et al., 2017), that a thorough systematic review should ideally include manual searches from multiple sources, such as expert consultations and grey literature searches. Although these elements were not comprehensively included in our methodology, we acknowledge this as a potential limitation of our study.

### Selection criteria

(1) Participants: preterm infants ≤34 weeks of gestation receiving respiratory support;
(2) Intervention groups: the iNO groups received standard treatments such as continuous positive airway pressure or intubation for respiratory support, combined with iNO therapy;
(3) Control groups: the control groups received standard treatment, with either nitrogen inhalation or none.
(4) Outcomes: the primary outcomes include death and/or BPD at 36 weeks of gestation. Follow-up studies also encompass assessments using the Bayley Mental Developmental Index (MDI), where the standardized mean motor score is 100 (SD 15). Scores below 85 indicate mild impairment, while scores below 70 signify moderate to severe impairment (Vohr et al., 2000). Additionally, assessments for CP or NDI are included. NDI is defined as any of the following: moderate-severe CP, blindness, deafness, or an MDI score lower than 70;
(5) Study type: RCTs.

### Study administration and data extraction

After duplicate studies were excluded by Endnote software, two reviewers (Z.F. and X.X.) independently selected eligible studies and extracted data. Disagreements were resolved by discussion and consensus by the reviewers. The extracted data included key elements such as the first author’s name, year of publication, sample size, and details about the patient population. Additionally, the data encompassed information regarding the geographic locations where the studies were conducted. It also specifically noted whether the studies were multicentre research projects. Detailed information about the time and dose variations of iNO, as well as the study outcomes, were also collected.

### Assessment of risk of bias and quality of evidence

Two reviewers (X.W. and Z.F.) independently assessed the data using the Cochrane ‘risk of bias assessment tool’ (first version) (Higgins et al., 2011). Assessment parameters included adequacy of random sequence generation, allocation concealment, blinding of assessors and caregivers, incomplete reporting of outcome data, selective reporting, and other biases. Each item was assessed as having a ‘low risk’, ‘unclear risk’, or ‘high risk’. Differences in the assessment of risk were resolved by discussion and based on more adequate justification. For each outcome, two authors (X.W. and X.X.) independently evaluated the quality of evidence using the Grading of Recommendations Assessment, Development and Evaluation (GRADE) method (Guyatt et al., 2011). The GRADE system accounts for eight aspects to determine the quality of evidence: risk of bias, inconsistency, indirectness, imprecision, publication bias, plus four additional factors: magnitude of effect, dose-response relationship, and absence of plausible confounding. Based on these criteria, the evidence quality was graded as high, moderate, low, or very low. Any disagreements were resolved through group consensus.

### Data analysis

Data analysis and graph production in this meta-analysis were performed by Review Manager 5.3. The effect size is expressed as risk ratios (RRs) with 95% confidence intervals (CIs). We extracted both crude and adjusted RR from the papers, and where necessary, transformed odds ratios (ORs) into RRs using established statistical methods (Grant, 2014). In instances where RR data were not explicitly provided in the articles, we calculated the RRs ourselves based on the incidence numbers and total population reported in the studies. For missing data, we endeavored to contact the authors of individual studies to acquire comprehensive information. We used fixed-effect meta-analysis to pool data when it was reasonable to assume that studies estimated the same underlying treatment effect (that is, trials were judged to be examining the same interventions and trial populations and methods were deemed sufficiently similar).

We estimated the treatment effects for each trial and examined intertrial heterogeneity through inspection of forest plots and use of the I^2^ statistic to quantify its impact: low (>25% and <50%), moderate (≥50% and <75%), or high (≥75%) (Higgins et al., 2003). Moreover, we implemented sensitivity and subgroup analyses to strengthen the robustness and applicability of our conclusions. Sensitivity analyses removed individual studies one-at-a-time to gauge the contribution of each one to our overall findings, boosting the reliability of our results. Additionally, subgroup analyses delved into the influence of various study-specific factors - such as patient demographics (age, exposure duration, starting dosage, maximum dosage, *etc.*) - affording deeper insights into nuanced effects across different study conditions.

Regarding publication bias, we conducted bias analysis for meta-analyses including more than ten studies. Tools like funnel plots were employed to detect possible publication biases, supplemented by Egger’s and Begg’s tests (Begg and Mazumdar, 1994; Egger et al., 1997). In cases where funnel plot asymmetry was detected, we applied the trim-and-fill method to adjust for potential biases (Duval and Tweedie, 2000), thereby enhancing the scientific robustness and reliability of our findings. A two-tailed *p*-value of less than 0.05 was considered statistically significant. Alongside assessing the statistical significance, we also aimed to discuss the clinical relevance of our findings, examining their potential impact on patient care and health outcomes.

## Results

### Study selection and characteristics of included studies

**FIGURE 1:**
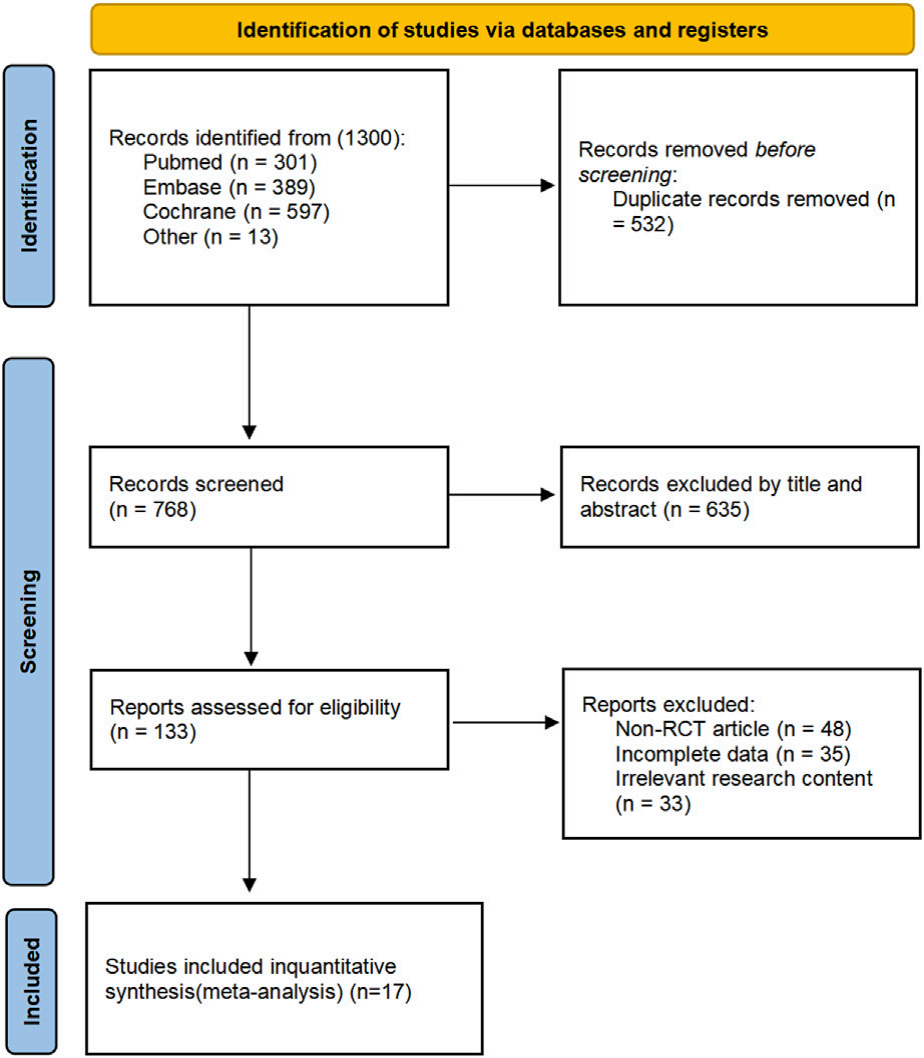
PRISMA study selection flowchart.

A total of 1,300 articles were obtained through the initial search and other sources. Next, 532 duplicate articles were excluded using software. Then, 635 articles were excluded by reading the title or abstract. Of the remaining 133 articles, 48 non-RCT studies, 35 with incomplete data, and 33 with irrelevant research content were excluded after full-text reading. Ultimately, 17 studies were included (Subhedar et al., 1997; Early compared, 1999; Kinsella et al., 1999; Srisuparp et al., 2002; Schreiber et al., 2003; Field et al., 2005; Hascoet et al., 2005; Van Meurs et al., 2005; Ballard et al., 2006; Dani et al., 2006; Kinsella et al., 2006; Van Meurs et al., 2007; Su and Chen, 2008; Mercier et al., 2010; Kinsella et al., 2014; Wei et al., 2014; Hasan et al., 2017). These studies primarily focused on outcomes such as BPD, mortality, and complications during hospitalization, which are the main focus of this meta-analysis. Together, these studies encompass a total of 4,080 neonates. The process of study selection is shown in Figure 1. Additionally, to analyze and investigate long-term neurological outcomes, including MDI scores below 70, CP, or NDI, this study also collected and analyzed 7 other follow-up studies (Bennett et al., 2001; Mestan et al., 2005; Hintz et al., 2007; Huddy et al., 2008; Watson et al., 2009; Walsh et al., 2010; Durrmeyer et al., 2013).

Thirteen studies included neonates within 3 days of birth (Early compared, 1999; Kinsella et al., 1999; Srisuparp et al., 2002; Schreiber et al., 2003; Hascoet et al., 2005; Van Meurs et al., 2005; Dani et al., 2006; Kinsella et al., 2006; Van Meurs et al., 2007; Su and Chen, 2008; Mercier et al., 2010; Kinsella et al., 2014; Wei et al., 2014), and the other four studies included neonates older than 3 days (Subhedar et al., 1997; Field et al., 2005; Ballard et al., 2006; Hasan et al., 2017). In nine trials (Kinsella et al., 1999; Field et al., 2005; Hascoet et al., 2005; Van Meurs et al., 2005; Kinsella et al., 2006; Van Meurs et al., 2007; Su and Chen, 2008; Mercier et al., 2010; Wei et al., 2014), the initial dose of iNO was 5 ppm, and 5 ppm was maintained during the study period in 3 of them (Kinsella et al., 1999; Kinsella et al., 2006; Mercier et al., 2010). In contrast, the maximum dose in 14 studies was ≥10 ppm, and the starting dose in 8 trials was ≥10 ppm (Subhedar et al., 1997; Early compared, 1999; Srisuparp et al., 2002; Schreiber et al., 2003; Ballard et al., 2006; Dani et al., 2006; Kinsella et al., 2014; Hasan et al., 2017). The duration of iNO was <7 days in nine studies (Subhedar et al., 1997; Srisuparp et al., 2002; Schreiber et al., 2003; Field et al., 2005; Hascoet et al., 2005; Van Meurs et al., 2005; Dani et al., 2006; Van Meurs et al., 2007; Su and Chen, 2008) and ≥7 days in seven studies (Kinsella et al., 1999; Ballard et al., 2006; Kinsella et al., 2006; Mercier et al., 2010; Kinsella et al., 2014; Wei et al., 2014; Hasan et al., 2017). The basic information of the included studies is shown in Table 1.

**TABLE 1:** Characteristics of included studies.

Study ID	Country; sites, n	Enrollment (iNO vs Control)	Gestational age; birth weight (g)	Enrollment age	Exposure time	Dose (start/end/maximum) (ppm)	Follow-up studies
Subhedar 1997 Subhedar et al. (1997)	United Kingdom; 1	42 (20: 22)	<32 weeks; 416–1,400 g	>4 days	3 days	20/5/20	Bennett 2001 Bennett et al. (2001)
Kinsella 1999 Kinsella et al. (1999)	US; 12	80 (48: 32)	<34 weeks	<7 days; 30 ± 38 h	7–14 days	5/5/5	__
NO trial group 1999 Early compared, (1999)	France; 33	192 (95: 97)	<33 weeks	<7 days; 1 (1, median)	__	10/20/20	__
Srisuparp 2002 Srisuparp et al. (2002)	US; 1	34 (16: 18)	<32 weeks; <2000 g	<3 days	3–7 days	20/0/20	__
Schreiber 2003 Schreiber et al. (2003)	US; 1	207 (105: 102)	<34 weeks; <2000 g	<3 days	<7 days	10/5/10	Mestan 2005 Mestan et al. (2005)
Van Meurs 2005 Van Meurs et al. (2005)	US; 16	420 (210: 210)	<34 weeks; 401–1,500 g	>4 h; mean 26–28 h	<14 days; 76 ± 73 h	5/10/10	HinZ 2007 Hintz et al. (2007)
Hascoet 2005 Hascoet et al. (2005)	France; 10	145 (61: 84)	<32 weeks	6 h–2 days	median: 28 h	5/10/10	__
Field 2005 Field et al. (2005)	United Kingdom; 15	108 (55: 53)	<34 weeks	<28 days	mean: 3.5 days	5/40/40	Huddy 2008 Huddy et al. (2008)
Dani 2006 Dani et al. (2006)	Italy; 1	40 (20: 20)	<30 weeks	≤7 days; 43.79 ± 25.0 h	3 days; 98.59 ± 21.4 h	10/6/10	__
Kinsella 2006 Kinsella et al. (2006)	US; 16	793 (395: 398)	≤34 weeks; 500–1,250 g	<2 days	21 days or until extubation	5/5/5	Watson 2009 Watson et al. (2009)
Ballard 2006 Ballard et al. (2006)	US; 21	582 (292: 288	≤32 weeks; 500–1,250 g	7 days–21 days	≥24 days	20/2/20	Walsh 2010 Walsh et al. (2010)
Van Meurs 2007 Van Meurs et al. (2007)	US; 1	29 (14: 15)	<34 weeks; >1,500 g	4 h–2 days	<14 days; 71.1 ± 30.9 h	5/10/10	__
Su 2008 Su and Chen, (2008)	China; 1	65 (32: 33)	<31 weeks; <1,500 g	<5 days; 2.45 ± 1.7 days	4.9 ± 2.3 days	5/20/20	__
Mercier 2010 Mercier et al. (2010)	US; 30	795 (399: 401)	>500 g	<24 h	7–21 days	5/5/5	Durrmeyer 2013 Durrmeyer et al. (2013)
Wei 2014 Wei et al. (2014)	China; 1	60 (30: 30)	26–34 weeks; 1,082–2,350 g	<7 days	>7 days or until extubation	5/-/10	__
Kinsella 2014 Kinsella et al. (2014)	US; 5	124 (59: 65)	≤34 weeks 500–1,250 g	<3 days	≥14 days	10/5/10	__
Hasan 2017 Hasan et al. (2017)	Canada; 33	451 (229: 222)	<30 weeks; <1,250 g	9.9 ± 2.8 days	24 days	20/5/20	__

### Assessment of risk of bias and quality of evidence

The details of randomization were described in 14 trials (Subhedar et al., 1997; Early compared, 1999; Kinsella et al., 1999; Schreiber et al., 2003; Field et al., 2005; Hascoet et al., 2005; Van Meurs et al., 2005; Ballard et al., 2006; Kinsella et al., 2006; Van Meurs et al., 2007; Mercier et al., 2010; Kinsella et al., 2014; Wei et al., 2014; Hasan et al., 2017), while the method of allocation concealment was reported in 14 trials (Subhedar et al., 1997; Early compared, 1999; Kinsella et al., 1999; Schreiber et al., 2003; Field et al., 2005; Hascoet et al., 2005; Van Meurs et al., 2005; Ballard et al., 2006; Dani et al., 2006; Kinsella et al., 2006; Van Meurs et al., 2007; Mercier et al., 2010; Kinsella et al., 2014; Hasan et al., 2017). Eight trials were rated as having a high risk of bias because participants and assessors were not blinded to outcomes (Subhedar et al., 1997; Early compared, 1999; Srisuparp et al., 2002; Field et al., 2005; Hascoet et al., 2005; Dani et al., 2006; Su and Chen, 2008; Wei et al., 2014). All attrition biases were rated as low risk due to continued follow-up of the study and no significant reduction in data loss. Regarding reporting bias, eight trials were rated as having an unclear risk of bias due to missing study registration (Subhedar et al., 1997; Early compared, 1999; Srisuparp et al., 2002; Schreiber et al., 2003; Hascoet et al., 2005; Dani et al., 2006; Su and Chen, 2008; Wei et al., 2014). Six of the other biases were rated as high risk, including three trials that were closed early due to adverse events, such as a high incidence of IVH or unclear therapeutic effects (Kinsella et al., 1999; Van Meurs et al., 2005; Dani et al., 2006) and three trials that were industry-funded (Subhedar et al., 1997; Schreiber et al., 2003; Mercier et al., 2010) (Supplementary Table S3). Overall, the quality of evidence ranges from moderate (such as NDI and pulmonary air leak) to high (such as BPD and/or death). The primary factors that reduce the quality of evidence are inconsistency (high heterogeneity between studies) and imprecision (wide 95% confidence intervals) (Supplementary Table S4).

### BPD at 36 weeks of gestation among survivors

**FIGURE 2:**
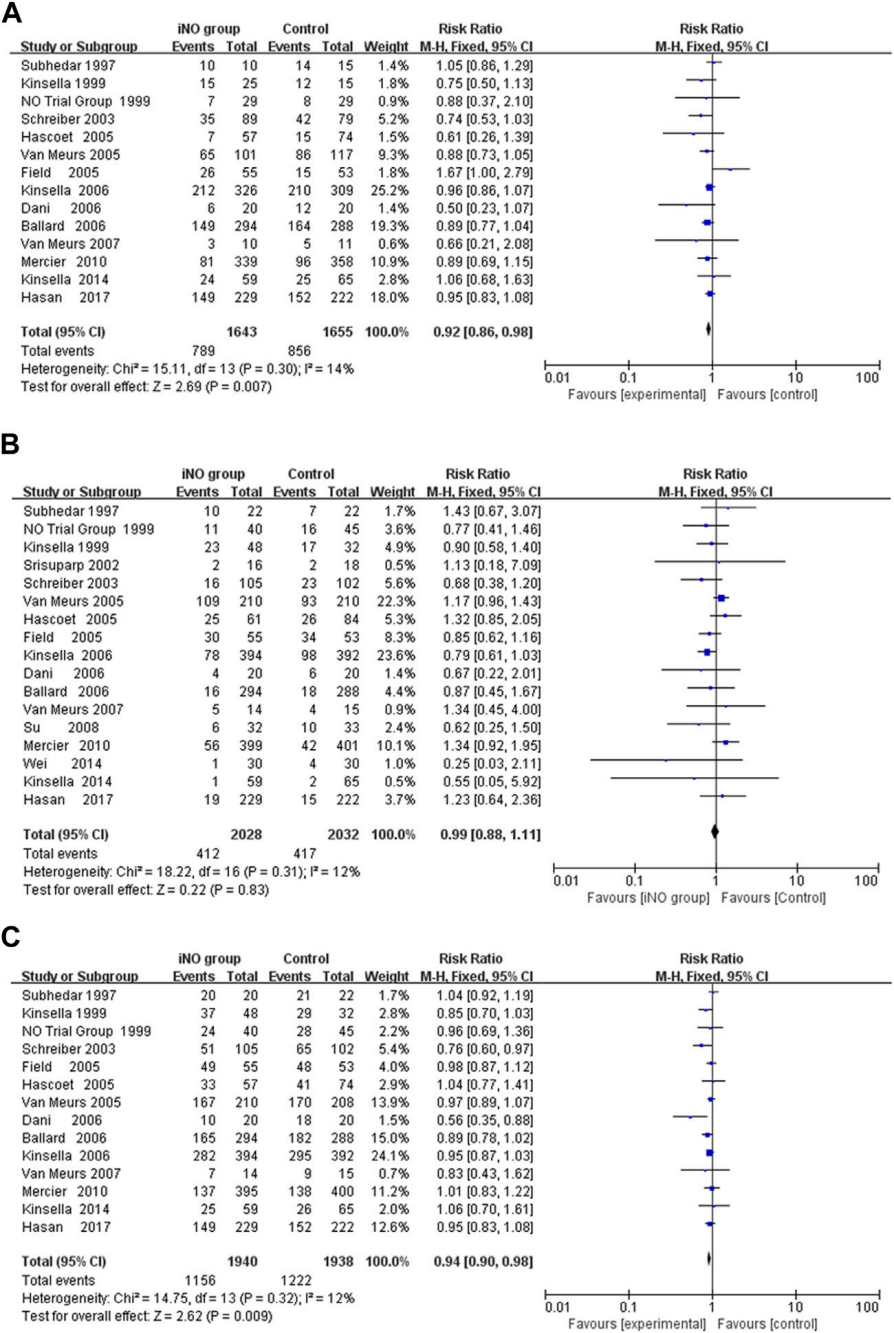
Forest plot comparing iNO group with control group in preterm infants; **(A)** BPD at 36 weeks of gestation, **(B)** Mortality during hospitalization, **(C)** Death or BPD at 36 weeks of gestation. BPD, bronchopulmonary dysplasia.

In the meta analyses of 14 studies (Subhedar et al., 1997; Early compared, 1999; Kinsella et al., 1999; Schreiber et al., 2003; Field et al., 2005; Hascoet et al., 2005; Van Meurs et al., 2005; Ballard et al., 2006; Dani et al., 2006; Kinsella et al., 2006; Van Meurs et al., 2007; Mercier et al., 2010; Kinsella et al., 2014; Hasan et al., 2017), we observed a significantly reduced incidence of BPD at 36 weeks of gestation in infants treated with NO compared to those receiving conventional respiratory support along with either nitrogen inhalation or none. This analysis used crude RR, not adjusted RR, showing a significant reduction in BPD incidence (789/1,643 vs 856/1,655; RR: 0.92; 95% CI: 0.86–0.98; I2 = 14%; *p* = 0.007) (Figure 2A). Sensitivity analysis conducted by sequentially excluding each trial did not significantly alter the results or heterogeneity (all *p* < 0.05, I^2^ < 25%). In order to delve deeper into the potential influences of age, birth weight, starting dose, and duration of iNO administration on neonates, we conducted a series of sub-group analyses. However, these did not reveal statistically significant differences among subgroups (*p* > 0.05). While no statistically significant differences between subgroups were found with a *p*-value above 0.05, other elements such as effect size and clinical significance should still be taken into account before reaching a definitive conclusion.

First, subgroup-analysis showed that iNO significantly reduced the incidence of BPD at 36 weeks of gestation in enrollment age ≤3 days (RR: 0.89; 95% CI: 0.83–0.96, I^2^ = 0%, *p* = 0.002) (Early compared, 1999; Kinsella et al., 1999; Schreiber et al., 2003; Hascoet et al., 2005; Van Meurs et al., 2005; Dani et al., 2006; Kinsella et al., 2006; Van Meurs et al., 2007; Mercier et al., 2010; Kinsella et al., 2014), but not in enrollment age >3 days (RR: 0.96; 95% CI: 0.87–1.05, I^2^ = 52%, *p* = 0.90) (Subhedar et al., 1997; Field et al., 2005; Ballard et al., 2006; Hasan et al., 2017). Next, a subgroup analysis of iNO duration <7 days failed to reduce the incidence of BPD at 36 weeks of gestation (RR: 0.87; 95% CI: 0.75–1.00, I^2^ = 56%, *p* = 0.06) (Subhedar et al., 1997; Schreiber et al., 2003; Field et al., 2005; Hascoet et al., 2005; Van Meurs et al., 2005; Dani et al., 2006; Van Meurs et al., 2007), while iNO with duration ≥7 days significantly reduced the rate of BPD at 36 weeks of gestation (RR: 0.93; 95% CI: 0.86–1.00, I^2^ = 0%, *p* = 0.04) (Kinsella et al., 1999; Ballard et al., 2006; Kinsella et al., 2006; Mercier et al., 2010; Kinsella et al., 2014; Hasan et al., 2017).

Neither the restricted iNO dose of 5 ppm (Kinsella et al., 1999; Kinsella et al., 2006; Mercier et al., 2010) nor the initial iNO dose of 5 ppm (Kinsella et al., 1999; Field et al., 2005; Hascoet et al., 2005; Van Meurs et al., 2005; Kinsella et al., 2006; Van Meurs et al., 2007; Mercier et al., 2010) could significantly change the incidence of BPD at 36 weeks of gestation (RR: 0.93, 0.93; 95% CI: 0.84–1.03, 0.85–1.02; I^2^ = 0%, 27%, *p* = 0.17, 0.13; respectively); however, the maximum dose of iNO ≥10 ppm (Subhedar et al., 1997; Early compared, 1999; Schreiber et al., 2003; Field et al., 2005; Hascoet et al., 2005; Van Meurs et al., 2005; Ballard et al., 2006; Dani et al., 2006; Van Meurs et al., 2007; Kinsella et al., 2014; Hasan et al., 2017) or the initial iNO dose ≥10 ppm (Subhedar et al., 1997; Schreiber et al., 2003; Ballard et al., 2006; Dani et al., 2006; Kinsella et al., 2014; Hasan et al., 2017) could reduce the incidence of BPD at 36 weeks of gestation (RR: 0.91, 0.90; 95% CI: 0.84–0.98, 0.82–0.98; I^2^ = 27%, 17%, *p* = 0.02, 0.02; respectively).

Finally, iNO did not significantly reduce the incidence of BPD at 36 weeks of gestation in preterm infants ≤1,000 g (RR: 0.96; 95% CI: 0.88–1.05, I^2^ = 26%, *p* = 0.25) (Schreiber et al., 2003; Van Meurs et al., 2005; Ballard et al., 2006; Kinsella et al., 2006; Kinsella et al., 2014), but in preterm infants >1,000 g (RR: 0.69; 95% CI: 0.53–0.90, I^2^ = 8%, *p* = 0.006) (Schreiber et al., 2003; Van Meurs et al., 2005; Kinsella et al., 2006; Kinsella et al., 2014) (Table 2).

**TABLE 2:** Subgroup analysis of BPD at 36 weeks of gestation among survivors.

Outcomes	Included studies	iNO group positive/total	Control group positive/total	Fixed/Random model	Heterogeneity (I^2^) (%)	RR (95% CI)	p	Test for subgroup difference (*p*-value)
Enrollment age								0.26
≤3d	10 Early compared, (1999); Kinsella et al. 1999; Schreiber et al. (2003); Hascoet et al. (2005); Van Meurs et al. (2005); Dani et al. (2006); Kinsella et al. (2006); Van Meurs et al. (2007); Mercier et al. (2010); Kinsella et al. (2014)	455/1,055	511/1,077	Fixed	0	0.89 (0.81–0.97)	0.007	
>3d	4 Subhedar et al. (1997); Field et al. (2005); Ballard et al. (2006); Hasan et al. (2017)	334/588	345/578	Fixed	52	0.96 (0.87–1.05)	0.90	
Exposure Time								0.39
<7 days	7 (Subhedar et al. (1997); Schreiber et al. (2003); Field et al. (2005); Hascoet et al. (2005); Van Meurs et al. (2005); Dani et al. (2006); Van Meurs et al. (2007)	152/342	189/369	Fixed	56	0.87 (0.75–1.00)	0.06	
≥7 days	6 (Kinsella et al. (1999); Ballard et al. (2006); Kinsella et al. (2006); Mercier et al. (2010); Kinsella et al. (2014); Hasan et al. (2017)	630/1,272	659/1,257	Fixed	0	0.93 (0.86–1.00)	0.04	
Starting dose								0.92
5 ppm	7 (Kinsella et al. (1999); Field et al. (2005); Hascoet et al. (2005); Van Meurs et al. (2005); Kinsella et al. (2006); Van Meurs et al. (2007); Mercier et al. (2010)	409/913	439/937	Fixed	27	0.93 (0.85–1.02)	0.13	
≥10 ppm	7 (Subhedar et al. (1997); Early compared, (1999); Schreiber et al. (2003); Ballard et al. (2006); Dani et al. (2006); Kinsella et al. (2014); Hasan et al. (2017)	380/730	417/718	Fixed	17	0.90 (0.82–0.98)	0.02	
Maximum dose during study								0.99
≤5 ppm	3 Kinsella et al. (1999); Kinsella et al. (2006); Mercier et al. (2010)	308/690	318/682	Fixed	0	0.93 (0.84–1.03)	0.17	
≥10 ppm	11 (Subhedar et al. (1997); Early compared, (1999); Schreiber et al. (2003); Field et al. (2005); Hascoet et al. (2005); Van Meurs et al. (2005); Ballard et al. (2006); Dani et al. (2006); Van Meurs et al. (2007); Kinsella et al. (2014); Hasan et al. (2017)	481/953	538/973	Fixed	27	0.91 (0.84–0.98)	0.02	
Birth weight								0.60
≤1,000 g	5 (Schreiber et al. (2003); Van Meurs et al. (2005); Ballard et al. (2006); Kinsella et al. (2006); Kinsella et al. (2014)	393/711	403/707	Fixed	26	0.96 (0.88–1.05)	0.25	
>1000 g	4 (Schreiber et al. (2003); Van Meurs et al. (2005); Kinsella et al. (2006); Kinsella et al. (2014)	58/186	81/176	Fixed	8	0.69 (0.53–0.90)	0.006	

BPD, bronchopulmonary dysplasia; CI, confidence interval; RR, risk ratio.

### Mortality during hospitalization

The researchers in all the studies provided mortality data, but there were differences in measures of survival or death, such as death at seven or 28 days after birth, 36 weeks of gestation, or before discharge. All data were collected during hospitalization, so we uniformly calculated the mortality rate during hospitalization. However, only Wei 2014 revealed that iNO reduced the incidence of mortality to a statistically significant level (RR: 0.25; 95% CI: 0.03–2.11). Meta-analyses of all RCTs showed that iNO failed to reduce the incidence of neonatal in-hospital mortality (412/2028 V S 417/2032; RR: 0.99; 95% CI: 0.88–1.11, I^2^ = 12%, *p* = 0.83) (Subhedar et al., 1997; Early compared, 1999; Kinsella et al., 1999; Srisuparp et al., 2002; Schreiber et al., 2003; Field et al., 2005; Hascoet et al., 2005; Van Meurs et al., 2005; Ballard et al., 2006; Dani et al., 2006; Kinsella et al., 2006; Van Meurs et al., 2007; Hasan et al., 2017) (Figure 2B). Similarly, iNO did not produce a significant difference in mortality in any of the subgroup analyses including enrollment age, exposure time, starting dose, maximum dose during study and birth weight (Supplementary Table S5).

### Death or BPD at 36 weeks of gestation

A meta-analysis of 14 RCTs showed that iNO produced a statistically significant difference in the incidence of BPD or death at 36 weeks of gestation in the intervention group compared to the control group (RR: 0.94; 95% CI: 0.90–0.98; I^2^ = 12%; *p* = 0.009) (Subhedar et al., 1997; Early compared, 1999; Kinsella et al., 1999; Schreiber et al., 2003; Field et al., 2005; Hascoet et al., 2005; Van Meurs et al., 2005; Ballard et al., 2006; Dani et al., 2006; Kinsella et al., 2006; Van Meurs et al., 2007; Mercier et al., 2010; Kinsella et al., 2014; Hasan et al., 2017) (Figure 2C). The heterogeneity test was performed by deleting each trial one by one, and the results did not produce significant changes. In subgroup analysis, iNO with dose ≥10 ppm (starting dose or maximum dose during study) significantly decreased composite outcome of BPD or mortality, and the effect was more pronounced in infants with birth weight ≥1,000 g (Supplementary Table S6).

### Short- and long-term neurological outcomes

**FIGURE 3:**
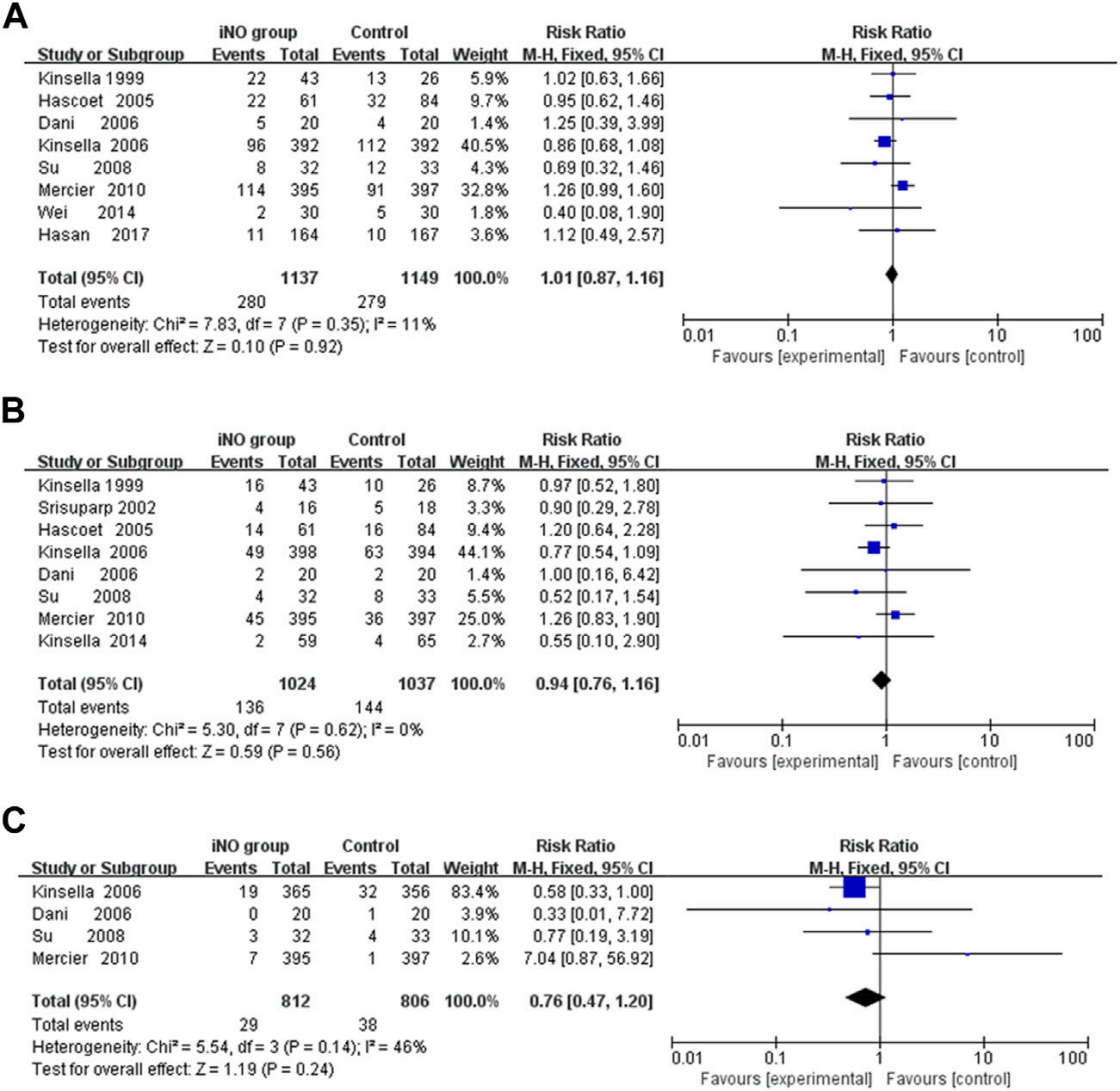
Forest plot comparison of short-term neural outcomes between iNO group and control group; **(A)** IVH (grade III or IV), **(B)** IVH (any grade), **(C)** PVL. IVH, intraventricular hemorrhage; PVL, periventricular leukomalacia.

Conducting a meta-analysis to investigate whether iNO increases the risk of IVH is worthwhile, particularly in light of research suggesting a link between NO and intracerebral hemorrhage (Li et al., 2011). IVH is graded based on the amount and location of brain hemorrhage. Grades I or II often have a better prognosis and are typically less severe, whereas Grades III or IV can lead to more serious neurological symptoms and even death. In the analysis, none of included studies showed that iNO increased the rate of IVH (any stage) (Kinsella et al., 1999; Hascoet et al., 2005; Dani et al., 2006; Kinsella et al., 2006; Su and Chen, 2008; Mercier et al., 2010; Wei et al., 2014; Hasan et al., 2017), IVH (grade III or IV) (Kinsella et al., 1999; Srisuparp et al., 2002; Hascoet et al., 2005; Dani et al., 2006; Kinsella et al., 2006; Su and Chen, 2008; Mercier et al., 2010; Kinsella et al., 2014) and PVL (Dani et al., 2006; Kinsella et al., 2006; Su and Chen, 2008; Mercier et al., 2010). In the meta-analysis, iNO also did not increase the incidence of IVH (any stage), IVH (grade III or IV) and PVL (RR: 1.01, 0.94, 0.76; 95% CI: 0.78–1.16, 0.76–1.16, 0.47–1.20; I^2^ = 11%, 0%, 46%; *p* = 0.92, 0.56, 0.24; respectively) (Figures 3A–C).

**FIGURE 4:**
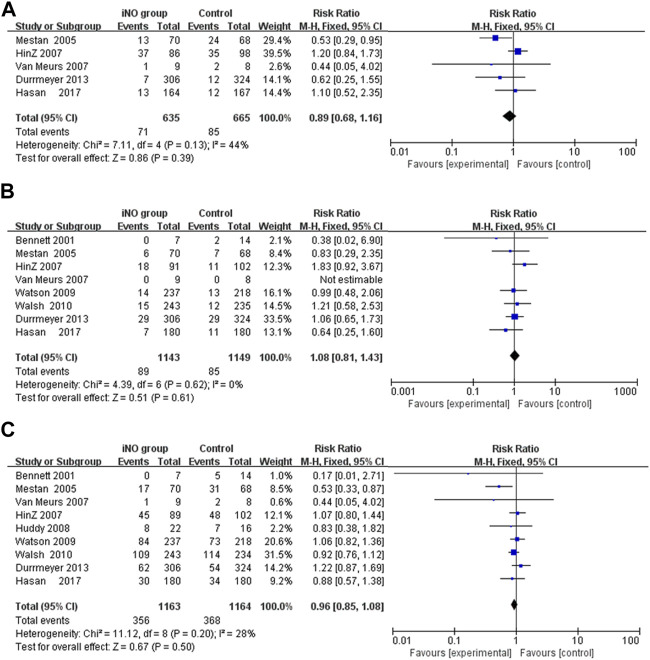
Comparison of long-term neurological outcomes between intervention and control groups in forest plots; **(A)** Bayley MDI <70, **(B)** CP, **(C)** NDI. CP, cerebral palsy; MDI, mental developmental index; NDI, neurodevelopmental impairment.

Among the long-term neurological outcomes, meta-analyses showed that iNO did not lead to poor long-term neurological outcomes (including Bayley MDI <70, CP or NDI) (RR: 0.89, 1.08, 0.96; CI: 0.68–1.16, 0.81–1.43, 0.85–1.08; I^2^ = 44%, 0%, 28%; *p* = 0.39, 0.61, 0.50; 5 trials (Mestan et al., 2005; Hintz et al., 2007; Van Meurs et al., 2007; Durrmeyer et al., 2013; Hasan et al., 2017), 8 trials (Bennett et al., 2001; Mestan et al., 2005; Hintz et al., 2007; Van Meurs et al., 2007; Watson et al., 2009; Walsh et al., 2010; Durrmeyer et al., 2013; Hasan et al., 2017), 9 trials (Bennett et al., 2001; Mestan et al., 2005; Hintz et al., 2007; Van Meurs et al., 2007; Huddy et al., 2008; Watson et al., 2009; Walsh et al., 2010; Durrmeyer et al., 2013; Hasan et al., 2017); respectively) (Figures 4A–C).

### Other outcomes

Other neonatal complications, including NEC, symptomatic PDA, sepsis, pulmonary hemorrhage, retinopathy of prematurity (ROP) requiring treatment, and pulmonary air leak (pneumothorax or interstitial emphysema), were also assessed. The meta-analysis of each complication showed that there was no significant difference between the iNO intervention group and the control group (Table 3).

**TABLE 3:** Neonatal outcomes.

Outcomes	Included studies	iNO group positive/total	Control group positive/total	Heterogeneity (I^2^) (%)	Fixed/Random model	RR (95% CI)	p
BPD at 36 weeks of gestation among survivors	14 (Subhedar et al. (1997); Early compared, (1999); Kinsella et al. (1999); Schreiber et al. (2003); Field et al. (2005); Hascoet et al. (2005); Van Meurs et al. (2005); Ballard et al. (2006); Dani et al. (2006); Kinsella et al. (2006); Van Meurs et al. (2007); Mercier et al. (2010); Kinsella et al. (2014); Hasan et al. (2017)	789/1,643	856/1,655	14	Fixed	0.92(0.86–0.98)	0.007
Mortality during hospitalization	17 (Subhedar et al. (1997); Early compared, (1999); Kinsella et al. (1999); Schreiber et al. (2003); Field et al. (2005); Hascoet et al. (2005); Van Meurs et al. (2005); Ballard et al. (2006); Dani et al. (2006); Kinsella et al. (2006); Van Meurs et al. (2007); Mercier et al. (2010); Kinsella et al. (2014); Hasan et al. (2017)	412/2028	417/2032	12	Fixed	0.99 (0.88–1.11)	0.83
Death or BPD at 36 weeks of gestation	14 (Subhedar et al. (1997); Early compared, (1999); Kinsella et al. (1999); Schreiber et al. (2003); Field et al. (2005); Hascoet et al. (2005); Van Meurs et al. (2005); Ballard et al. (2006); Dani et al. (2006); Kinsella et al. (2006); Van Meurs et al. (2007); Mercier et al. (2010); Kinsella et al. (2014); Hasan et al. (2017)	1,156/1940	1,222/1938	12	Fixed	0.94 (0.90–0.98)	0.009
IVH (grade III or IV)	8 (Kinsella et al. (1999); Srisuparp et al. (2002); Hascoet et al. (2005); Dani et al. (2006); Kinsella et al. (2006); Su and Chen, (2008); Mercier et al. (2010); Kinsella et al. (2014)	136/1,024	144/1,037	0	Fixed	0.94(0.76–1.16)	0.56
IVH (any grade)	8 (Kinsella et al. (1999); Hascoet et al. (2005); Dani et al. (2006); Kinsella et al. (2006); Su and Chen, (2008); Mercier et al. (2010); Wei et al. (2014); Hasan et al. (2017)	280/1,137	279/1,149	11	Fixed	1.01 (0.87–1.16)	0.92
PVL	4 (Dani et al. (2006); Kinsella et al. (2006); Su and Chen, (2008); Mercier et al. (2010)	29/812	38/806	46	Fixed	0.76 (0.47–1.20)	0.24
Bayley MDI <70	5 (Mestan et al. (2005); Hintz et al. (2007); Van Meurs et al. (2007); Durrmeyer et al. (2013); Hasan et al. (2017)	71/635	85/665	44	Fixed	0.89 (0.68–1.16)	0.39
CP	8 (Bennett et al. (2001); Mestan et al. (2005); Hintz et al. (2007); Van Meurs et al. (2007); Watson et al. (2009); Walsh et al. (2010); Durrmeyer et al. (2013); Hasan et al. (2017)	89/1,143	85/1,149	0	Fixed	1.08 (0.81–1.43)	0.61
NDI	9 (Bennett et al. (2001); Mestan et al. (2005); Hintz et al. (2007); Van Meurs et al. (2007); Huddy et al. (2008); Watson et al. (2009); Walsh et al. (2010); Durrmeyer et al. (2013); Hasan et al. (2017)	356/1,163	368/1,164	28	Fixed	0.96 (0.85–1.08)	0.50
NEC	12 (Subhedar et al. (1997); Srisuparp et al. (2002); Schreiber et al. (2003); Hascoet et al. (2005); Ballard et al. (2006); Dani et al. (2006); Kinsella et al. (2006); Hintz et al. (2007); Su and Chen, (2008); Mercier et al. (2010); Kinsella et al. (2014); Hasan et al. (2017)	139/1700	123/1,499	0	Fixed	1.18 (0.92–1.51)	0.20
Symptomatic PDA	11 (Subhedar et al. 1997; Srisuparp et al. 2002; Schreiber et al. 2003; Field et al. 2005; Hascoet et al. 2005; Ballard et al. 2006; Kinsella et al. 2006; Su and Chen, 2008; Mercier et al. 2010; Kinsella et al. 2014; Hasan et al. 2017)	453/1,664	460/1,679	0	Fixed	0.99 (0.90–1.10)	0.91
Sepsis	12 (Subhedar et al. (1997); Srisuparp et al. (2002); Schreiber et al. (2003); Field et al. (2005); Van Meurs et al. (2005); Ballard et al. (2006); Dani et al. (2006); Kinsella et al. (2006); Su and Chen, (2008); Mercier et al. (2010); Kinsella et al. (2014); Hasan et al. (2017)	442/1816	413/1799	0	Fixed	1.05 (0.94–1.17)	0.38
Pulmonary hemorrhage	10 (Subhedar et al. (1997); Srisuparp et al. (2002); Schreiber et al. (2003); Field et al. (2005); Van Meurs et al. (2005); Kinsella et al. (2006); Van Meurs et al. (2007); Su and Chen, (2008); Mercier et al. (2010); Wei et al. (2014)	72/1,275	73/1,275	0	Fixed	0.98 (0.72–1.35)	0.92
ROP requiring treatment	9 (Subhedar et al. (1997); Schreiber et al. (2003); Field et al. (2005); Van Meurs et al. (2005); Ballard et al. (2006); Kinsella et al. (2006); Van Meurs et al. (2007); Su and Chen, (2008); Kinsella et al. (2014)	187/1,066	193/1,075	0	Fixed	0.98 (0.82–1.18)	0.85
Pulmonary air leak	11 (Subhedar et al. (1997); Srisuparp et al. (2002); Schreiber et al. (2003); Field et al. (2005); Van Meurs et al. (2005); Kinsella et al. (2006); Van Meurs et al. (2007); Su and Chen, (2008); Mercier et al. (2010); Wei et al. (2014); Hasan et al. (2017)	113/1,504	128/1,497	0	Fixed	0.88 (0.69–1.11)	0.27

BPD, bronchopulmonary dysplasia; CI, confidence interval; CP, cerebral palsy; MDI, mental developmental index; NDI, neurodevelopmental impairment, i.e, any of the following: moderate-severe CP, blind, deaf, MDI < 70; NEC, necrotizing enterocolitis; PDA, patent ductus arteriosus; IVH, intraventricular hemorrhage; PVL, periventricular leukomalacia; ROP, retinopathy of prematurity; RR, risk ratio.

### Publication bias

**FIGURE 5:**
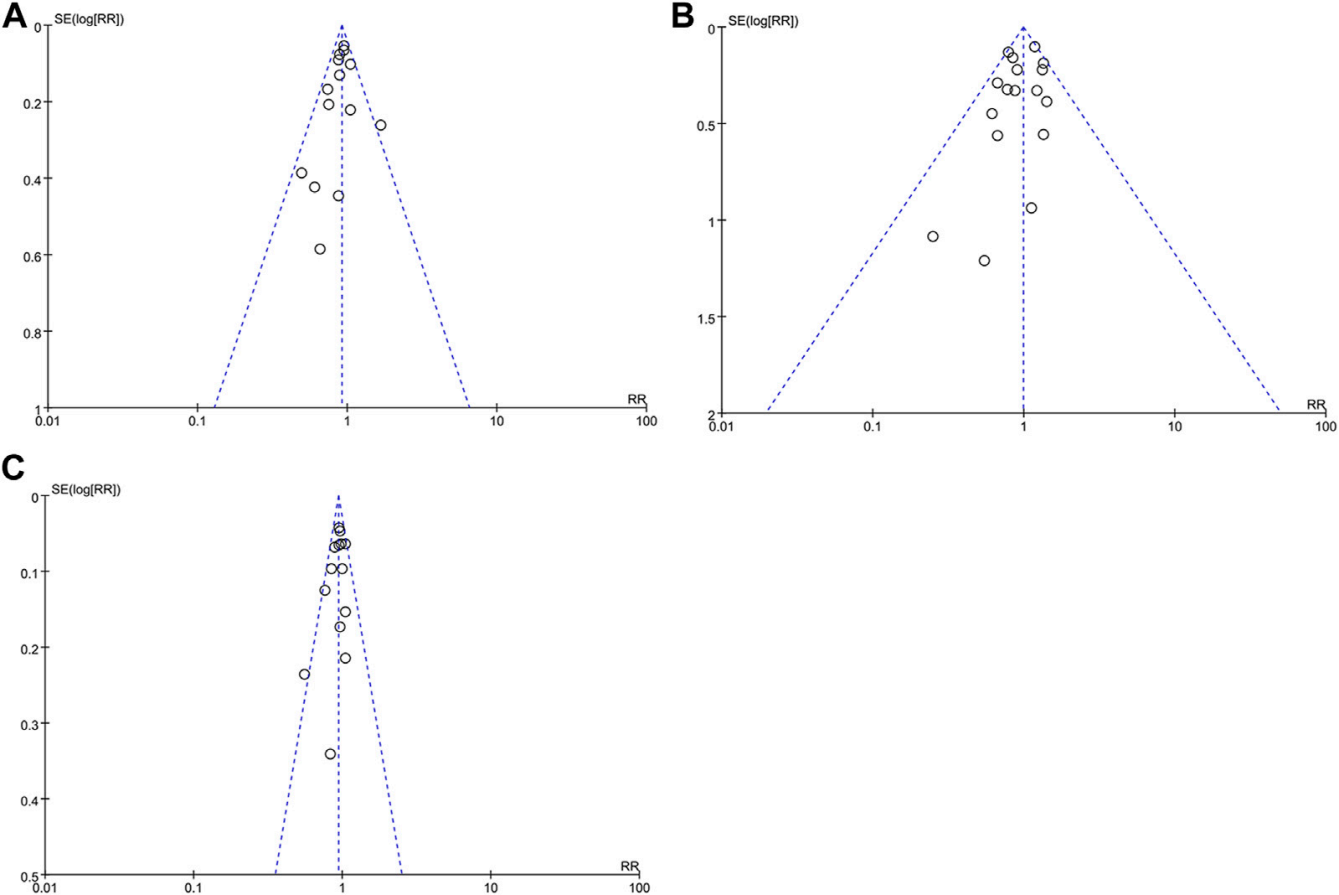
Funnel plot with estimated 95% CI for meta-analysis of the effect of iNO treatment on BPD at 36 weeks of gestation among survivors **(A)**, mortality during hospitalization **(B)**, and death or BPD at 36 weeks of gestation **(C)**. BPD, bronchopulmonary dysplasia; CI, confidence intervals; RR, risk ratio.

Funnel plots of the composite outcome of BPD at 36 weeks of gestation among survivors, mortality during hospitalization, and death or BPD at 36 weeks of gestation were symmetrical (Figures 5A–C), indicating no significant sources of publication bias confirmed by both Begg rank correlation method and Egger weighted regression test (*p* > 0.05).

## Discussion

Considering the above meta-analyses together, we found that iNO reduced the incidence of BPD and the composite outcome of BPD or death without increasing the incidence of short-term or long-term neurological disorders (IVH, PVL, CP and NDI) or leading to other short-term outcomes, such as ROP requiring treatment, NEC, sepsis, pulmonary leak, pulmonary hemorrhage, and symptomatic PDA. Subgroup analysis indicated that a more intensive approach to iNO treatment might be beneficial. This entails initiating iNO therapy earlier in neonates (≤3 days of age), applying it to infants with a birth weight over 1,000 g, extending the treatment duration to more than 7 days, and using a higher dosage of iNO (at least 10 ppm). These strategies have been found to be more effective in reducing the incidence of BPD according to our findings.

Although a more aggressive application of iNO was found to correspond with a more favorable outcome in neonates in this study, the side effects of NO need our attention. NO can potentially cause oxidative stress. Researchers have reported a high risk of neonatal methemoglobinemia after being treated with high concentrations of inhaled NO (up to 80 ppm) (Neonatal Inhaled Nitric Oxide Study, 1997; Roberts et al., 1997; Davidson et al., 1998), but the incidence was significantly lower in children after being treated with low concentrations of inhaled NO (Hamon et al., 2010; Rhee et al., 2013). Ballard RA et al. reported that preterm infants with a birth weight <1,250 g received iNO treatment at a dose starting from 20 ppm for at least 24 days, and no methemoglobinemia occurred, indicating that low concentrations of iNO did not cause methemoglobinemia (Ballard et al., 2006). To better avoid the disadvantages of iNO, routine monitoring of methemoglobin in premature infants treated with iNO is recommended. Additionally, the bleeding risk associated with NO warrant attention. In previous studies, it has been suggested that NO can inhibit platelet aggregation, leading to prolonged bleeding time and increased bleeding tendency (Hogman et al., 1994; Cheung et al., 1997; George et al., 1998). According to the National Institutes of Health, it is not recommended to administer iNO therapy in preterm infants due to the imprecise efficacy of iNO in preterm infants and its potential to inhibit platelet aggregation and increase the risk of IVH (Cole et al., 2011). In addition, the current regulatory status of NO in both the EU and US is noteworthy. As per the U.S. Food and Drug Administration, NO is indicated for term and near-term (>34 weeks of gestation) neonates with hypoxic respiratory failure and evidence of pulmonary hypertension, in conjunction with ventilatory support and other agents. However, its efficacy and safety in premature neonates for the prevention of BPD have not shown substantial evidence (FDA, 2019). Similarly, the European Medicines Agency’s label states that the safety and efficacy of iNO in premature infants less than 34 weeks gestation have not yet been established, with no recommendation or posology provided for this population (Agency, 2022). The results of this meta-analysis showed that iNO did not increase the incidence of bleeding diseases such as pulmonary hemorrhage and IVH. This result is consistent with that reported in other meta-analyses (Donohue et al., 2011; Barrington et al., 2017b). In addition, in terms of laboratory tests, Thom et al. analyzed the trend of platelet count in infants with pulmonary arterial hypertension who received iNO treatment, and the results showed that during iNO treatment, the platelet count remained basically stable (Thom et al., 2020).

In a series of clinical studies, the concentration of iNO mostly used was 5–40 ppm, but there was no definite conclusion about the relationship between iNO dose and response. A multicenter study showed that there was no difference in the clinical efficacy of different doses (5 ppm, 20 ppm, or 80 ppm) of iNO in the treatment of persistent pulmonary hypertension, and 5 or 20 ppm of iNO was safe and effective (Davidson et al., 1998). However, for BPD, none of the included studies found that the dose of iNO had a significant effect on its incidence, whereas the meta-analysis showed that iNO could reduce its incidence. Neither the initial dose nor the maintenance dose of 5 ppm plays a positive role in the prevention or treatment of BPD, and only ≥10 ppm has a significant effect. This result is similar to that obtained by Donohue PK et al., who included four RCTs with a maximum dose of 10 ppm in their meta-analysis (Donohue et al., 2011). Additionally, 2 multicenter nonrandomized studies by Chinese scholars did not show that a dose of inhaled NO ≤ 5 ppm had a significant effect on the incidence of BPD (Wang et al., 2011; Jiang et al., 2016). Therefore, iNO therapy with an initial dose starting from 10 ppm in preterm infants with a gestational age <34 weeks can be considered.

NO is one of the important substances required for normal lung development. In neonates with asphyxia, pneumonia or immature lung development, the lung cannot generate enough NO for physiological needs, so the pulmonary arterioles constrict, resulting in pulmonary hypertension and hypoxic respiratory failure (Hinton et al., 2006). Hypoxia, in turn, exacerbates lung damage, creating a vicious cycle. Inhalation of exogenous NO can increase the blood flow of the lungs with a better ventilation effect to improve oxygenation and inhibit inflammation. Early postnatal administration of iNO can increase pulmonary surfactant activity, promote pulmonary angiogenesis and alveolarization, reduce airway resistance, and prevent lung injury (McCurnin et al., 2005). This finding can also be assessed to explain why NO intervention in the early neonatal period (<3 days) is more effective in preventing BPD. Therefore, early administration of iNO may reduce the incidence of adverse clinical outcomes of premature infants.

Unfortunately, none of the included RCTs showed that iNO reduced the incidence of mortality; no significant difference was found in the final results of the meta-analysis, regardless of whether it was preterm infants with a body weight greater than or less than 1,000 g. However, the incidence of BPD was reduced. Although the improvement effect is not as good as that of the application of glucocorticoids or pulmonary surfactant, it is also a potentially effective means for BPD prevention. In addition, in the Ballard et al. study, the economic benefits were evaluated and although iNO has additional treatment costs, it shortens the length of hospital stay and reduces the cost of hospitalization, thus increasing the economic benefits (Zupancic et al., 2009). Therefore, for better treatment, iNO can be applied to premature infants as appropriate to improve short-term oxygenation, reduce the incidence of chronic lung diseases, and reduce the economic burden on patients.

Despite our efforts to conduct a comprehensive and accurate meta-analysis, several limitations remain. First, due to differences in gestational age and birth weight, lung tissue at different developmental stages responds differently to NO, which may affect the incidence of BPD, pulmonary hemorrhage, or other complications. Second, other baseline data, such as intervention dose and exposure time, as well as the sex of the infants included, were not uniform among the included trials, which may also have some impact on the results. At present, due to the limited number of studies, it is difficult to evaluate the interference factors of each group more accurately, therefore more large-sample, multicenter clinical studies are needed to further explore the efficacy and safety of iNO.

## Conclusion

In summary, the clinical application of iNO has been demonstrated to significantly reduce the incidence of BPD at 36 weeks of gestation. This reduction is contingent on several factors, including the infant’s age, birth weight, as well as the duration and dosage of iNO administration. iNO can be applied as an adjunctive therapy for hypoxia and as a preventive strategy for BPD in premature infants. It is recommended to consider initiating iNO treatment at a dosage of 10 ppm, especially in preterm infants with a gestational age of less than 34 weeks. Moving forward, it is imperative to conduct larger, multicenter clinical studies to further investigate the efficacy and safety of iNO, enhancing our understanding and application of this treatment in neonatal care.

## Abbreviations

BPD, bronchopulmonary dysplasia; CI, confidence interval; CP, cerebral palsy; iNO, inhaled nitric oxide; IVH, intraventricular hemorrhage; MDI, mental developmental index; NDI, neurodevelopmental impairment; NEC, necrotizing enterocolitis; PDA, patent ductus arteriosus; PVL, periventricular leukomalacia; RCT, randomized controlled trial; ROP, retinopathy of prematurity; RR, risk ratio.

## References

[B1] Agency (2022). INOmax EPAR product information. Available at: www.ema.europa.eu/en/documents/product-information/inomax-epar-product-information_en.pdf.

[B2] Ballard R. A. Truog W. E. Cnaan A. Martin R. J. Ballard P. L. Merrill J. D. (2006). Inhaled nitric oxide in preterm infants undergoing mechanical ventilation. N. Engl. J. Med. 355, 343–353. 10.1056/NEJMoa061088 16870913

[B3] Barrington K. J. Finer N. Pennaforte T. (2017b). Inhaled nitric oxide for respiratory failure in preterm infants. Cochrane Database Syst. Rev. 1, CD000509. 10.1002/14651858.CD000509 28045472 PMC6464861

[B4] Barrington K. J. Finer N. Pennaforte T. Altit G. (2017a). Nitric oxide for respiratory failure in infants born at or near term. Cochrane Database Syst. Rev. 1, CD000399. 10.1002/14651858.CD000399 28056166 PMC6464941

[B5] Begg C. B. Mazumdar M. (1994). Operating characteristics of a rank correlation test for publication bias. Biometrics 50, 1088–1101. 10.2307/2533446 7786990

[B6] Bennett A. J. Shaw N. J. Gregg J. E. Subhedar N. V. (2001). Neurodevelopmental outcome in high-risk preterm infants treated with inhaled nitric oxide. Acta Paediatr. 90, 573–576. 10.1111/j.1651-2227.2001.tb00801.x 11430720

[B7] Cannavò L. Perrone S. Viola V. Marseglia L. Di Rosa G. Gitto E. (2021). Oxidative stress and respiratory diseases in preterm newborns. Int. J. Mol. Sci., 22. 10.3390/ijms222212504 34830385 PMC8625766

[B8] Cheung P. Y. Salas E. Schulz R. Radomski M. W. (1997). Nitric oxide and platelet function: implications for neonatology. Semin. Perinatol. 21, 409–417. 10.1016/s0146-0005(97)80006-7 9352613

[B9] Cole F. S. Alleyne C. Barks J. D. Boyle R. J. Carroll J. L. Dokken D. (2011). NIH Consensus Development Conference statement: inhaled nitric-oxide therapy for premature infants. Pediatrics 127, 363–369. 10.1542/peds.2010-3507 21220405

[B10] Dani C. Bertini G. Pezzati M. Filippi L. Cecchi A. Rubaltelli F. F. (2006). Inhaled nitric oxide in very preterm infants with severe respiratory distress syndrome. Acta Paediatr. 95, 1116–1123. 10.1080/08035250600702594 16938760

[B11] Dani C. Corsini I. Cangemi J. Vangi V. Pratesi S. (2017). Nitric oxide for the treatment of preterm infants with severe RDS and pulmonary hypertension. Pediatr. Pulmonol. 52, 1461–1468. 10.1002/ppul.23843 29058384

[B12] Davidson D. Barefield E. S. Kattwinkel J. Dudell G. Damask M. Straube R. (1998). Inhaled nitric oxide for the early treatment of persistent pulmonary hypertension of the term newborn: a randomized, double-masked, placebo-controlled, dose-response, multicenter study. The I-NO/PPHN Study Group. Pediatrics 101, 325–334. 10.1542/peds.101.3.325 9480993

[B13] Donohue P. K. Gilmore M. M. Cristofalo E. Wilson R. F. Weiner J. Z. Lau B. D. (2011). Inhaled nitric oxide in preterm infants: a systematic review. Pediatrics 127, e414–e422. 10.1542/peds.2010-3428 21220391

[B14] Durrmeyer X. Hummler H. Sanchez-Luna M. Carnielli V. P. Field D. Greenough A. (2013). Two-year outcomes of a randomized controlled trial of inhaled nitric oxide in premature infants. Pediatrics 132, e695–e703. 10.1542/peds.2013-0007 23940237

[B15] Duval S. Tweedie R. (2000). Trim and fill: a simple funnel-plot-based method of testing and adjusting for publication bias in meta-analysis. Biometrics 56, 455–463. 10.1111/j.0006-341x.2000.00455.x 10877304

[B16] Early compared (1999). Early compared with delayed inhaled nitric oxide in moderately hypoxaemic neonates with respiratory failure: a randomised controlled trial. The Franco-Belgium Collaborative NO Trial Group. Lancet 354, 1066–1071. 10509497

[B17] Egger M. Davey Smith G. Schneider M. Minder C. (1997). Bias in meta-analysis detected by a simple, graphical test. BMJ 315, 629–634. 10.1136/bmj.315.7109.629 9310563 PMC2127453

[B18] FDA (2019). (nitric oxide) gas, for inhalation. USA: US Food and Drug Administration. Available at: www.accessdata.fda.gov/drugsatfda_docs/label/2019/020845s020lbl.pdf.

[B19] Field D. Elbourne D. Truesdale A. Grieve R. Hardy P. Fenton A. C. (2005). Neonatal ventilation with inhaled nitric oxide versus ventilatory support without inhaled nitric oxide for preterm infants with severe respiratory failure: the INNOVO multicentre randomised controlled trial (ISRCTN 17821339). Pediatrics 115, 926–936. 10.1542/peds.2004-1209 15805366

[B20] Forstermann U. Sessa W. C. (2012). Nitric oxide synthases: regulation and function. Eur. Heart J. 33, 829–837. 37a-37d. 10.1093/eurheartj/ehr304 21890489 PMC3345541

[B21] George T. N. Johnson K. J. Bates J. N. Segar J. L. (1998). The effect of inhaled nitric oxide therapy on bleeding time and platelet aggregation in neonates. J. Pediatr. 132, 731–734. 10.1016/s0022-3476(98)70370-1 9580780

[B22] Goldman A. P. Tasker R. C. Haworth S. G. Sigston P. E. Macrae D. J. (1996). Four patterns of response to inhaled nitric oxide for persistent pulmonary hypertension of the newborn. Pediatrics 98, 706–713. 10.1542/peds.98.4.706 8885950

[B23] Grant R. L. (2014). Converting an odds ratio to a range of plausible relative risks for better communication of research findings. Bmj 348, f7450. 10.1136/bmj.f7450 24464277

[B24] Guyatt G. Oxman A. D. Akl E. A. Kunz R. Vist G. Brozek J. (2011). GRADE guidelines: 1. Introduction-GRADE evidence profiles and summary of findings tables. J. Clin. Epidemiol. 64, 383–394. 10.1016/j.jclinepi.2010.04.026 21195583

[B25] Hamon I. Gauthier-Moulinier H. Grelet-Dessioux E. Storme L. Fresson J. Hascoet J. M. (2010). Methaemoglobinaemia risk factors with inhaled nitric oxide therapy in newborn infants. Acta Paediatr. 99, 1467–1473. 10.1111/j.1651-2227.2010.01854.x 20456277

[B26] Hasan S. U. Potenziano J. Konduri G. G. Perez J. A. Van Meurs K. P. Walker M. W. (2017). Effect of inhaled nitric oxide on survival without bronchopulmonary dysplasia in preterm infants: a randomized clinical trial. JAMA Pediatr. 171, 1081–1089. 10.1001/jamapediatrics.2017.2618 28973344 PMC5710365

[B27] Hascoet J. M. Fresson J. Claris O. Hamon I. Lombet J. Liska A. (2005). The safety and efficacy of nitric oxide therapy in premature infants. J. Pediatr. 146, 318–323. 10.1016/j.jpeds.2004.10.019 15756211

[B28] Higgins J. P. Altman D. G. Gotzsche P. C. Juni P. Moher D. Oxman A. D. (2011). The Cochrane Collaboration's tool for assessing risk of bias in randomised trials. BMJ 343, d5928. 10.1136/bmj.d5928 22008217 PMC3196245

[B29] Higgins J. P. Thompson S. G. Deeks J. J. Altman D. G. (2003). Measuring inconsistency in meta-analyses. Bmj 327, 557–560. 10.1136/bmj.327.7414.557 12958120 PMC192859

[B30] Hinton M. Mellow L. Halayko A. J. Gutsol A. Dakshinamurti S. (2006). Hypoxia induces hypersensitivity and hyperreactivity to thromboxane receptor agonist in neonatal pulmonary arterial myocytes. Am. J. Physiol. Lung Cell. Mol. Physiol. 290, L375–L384. 10.1152/ajplung.00307.2005 16214814

[B31] Hintz S. R. Van Meurs K. P. Perritt R. Poole W. K. Das A. Stevenson D. K. (2007). Neurodevelopmental outcomes of premature infants with severe respiratory failure enrolled in a randomized controlled trial of inhaled nitric oxide. J. Pediatr. 151 (16-22), 16–22.e3. 10.1016/j.jpeds.2007.03.017 17586184 PMC2770191

[B32] Hogman M. Frostell C. Arnberg H. Sandhagen B. Hedenstierna G. (1994). Prolonged bleeding time during nitric oxide inhalation in the rabbit. Acta Physiol. Scand. 151, 125–129. 10.1111/j.1748-1716.1994.tb09728.x 8048332

[B33] Huddy C. L. Bennett C. C. Hardy P. Field D. Elbourne D. Grieve R. (2008). The INNOVO multicentre randomised controlled trial: neonatal ventilation with inhaled nitric oxide versus ventilatory support without nitric oxide for severe respiratory failure in preterm infants: follow up at 4-5 years. Arch. Dis. Child. Fetal Neonatal Ed. 93, F430–F435. 10.1136/adc.2007.129353 18375612

[B35] Jiang Q. Gao X. Liu C. Chen D. Lin X. Xia S. (2016). Early inhaled nitric oxide in preterm infants <34 weeks with evolving bronchopulmonary dysplasia. J. Perinatol. 36, 883–889. 10.1038/jp.2016.112 27442155

[B36] Kinsella J. P. Cutter G. R. Steinhorn R. H. Nelin L. D. Walsh W. F. Finer N. N. (2014). Noninvasive inhaled nitric oxide does not prevent bronchopulmonary dysplasia in premature newborns. J. Pediatr. 165, 1104–1108. 10.1016/j.jpeds.2014.06.018 25063725 PMC4464845

[B37] Kinsella J. P. Cutter G. R. Walsh W. F. Gerstmann D. R. Bose C. L. Hart C. (2006). Early inhaled nitric oxide therapy in premature newborns with respiratory failure. N. Engl. J. Med. 355, 354–364. 10.1056/NEJMoa060442 16870914

[B38] Kinsella J. P. Walsh W. F. Bose C. L. Gerstmann D. R. Labella J. J. Sardesai S. (1999). Inhaled nitric oxide in premature neonates with severe hypoxaemic respiratory failure: a randomised controlled trial. Lancet 354, 1061–1065. 10.1016/s0140-6736(99)03558-8 10509496

[B39] Krasuski R. A. Warner J. J. Wang A. Harrison J. K. Tapson V. F. Bashore T. M. (2000). Inhaled nitric oxide selectively dilates pulmonary vasculature in adult patients with pulmonary hypertension, irrespective of etiology. J. Am. Coll. Cardiol. 36, 2204–2211. 10.1016/s0735-1097(00)00994-3 11127462

[B40] Li N. Worthmann H. Deb M. Chen S. Weissenborn K. (2011). Nitric oxide (NO) and asymmetric dimethylarginine (ADMA): their pathophysiological role and involvement in intracerebral hemorrhage. Neurol. Res. 33, 541–548. 10.1179/016164111X13007856084403 21669125

[B41] Lin F. Dong H. Song Y. Zhang T. Qi J. Xiao X. (2017). Effect of bronchopulmonary dysplasia on early intellectual development in preterm infants. Pediatr. Int. 59, 691–697. 10.1111/ped.13257 28177185

[B42] Majnemer A. Riley P. Shevell M. Birnbaum R. Greenstone H. Coates A. L. (2000). Severe bronchopulmonary dysplasia increases risk for later neurological and motor sequelae in preterm survivors. Dev. Med. Child. Neurol. 42, 53–60. 10.1017/s001216220000013x 10665976

[B43] Mandell E. Kinsella J. P. Abman S. H. (2021). Persistent pulmonary hypertension of the newborn. Pediatr. Pulmonol. 56, 661–669. 10.1002/ppul.25073 32930508

[B44] McCurnin D. C. Pierce R. A. Chang L. Y. Gibson L. L. Osborne-Lawrence S. Yoder B. A. (2005). Inhaled NO improves early pulmonary function and modifies lung growth and elastin deposition in a baboon model of neonatal chronic lung disease. Am. J. Physiol. Lung Cell. Mol. Physiol. 288, L450–L459. 10.1152/ajplung.00347.2004 15591412 PMC11784793

[B45] McGoldrick E. Stewart F. Parker R. Dalziel S. R. (2020). Antenatal corticosteroids for accelerating fetal lung maturation for women at risk of preterm birth. Cochrane Database Syst. Rev. 12, CD004454. 10.1002/14651858.CD004454.pub4 33368142 PMC8094626

[B46] Mercier J. C. Hummler H. Durrmeyer X. Sanchez-Luna M. Carnielli V. Field D. (2010). Inhaled nitric oxide for prevention of bronchopulmonary dysplasia in premature babies (EUNO): a randomised controlled trial. Lancet 376, 346–354. 10.1016/S0140-6736(10)60664-2 20655106

[B47] Mestan K. K. Marks J. D. Hecox K. Huo D. Schreiber M. D. (2005). Neurodevelopmental outcomes of premature infants treated with inhaled nitric oxide. N. Engl. J. Med. 353, 23–32. 10.1056/NEJMoa043514 16000353

[B48] Neonatal Inhaled Nitric Oxide Study G. (1997). Inhaled nitric oxide in full-term and nearly full-term infants with hypoxic respiratory failure. N. Engl. J. Med. 336, 597–604. 10.1056/NEJM199702273360901 9036320

[B49] Page M. J. McKenzie J. E. Bossuyt P. M. Boutron I. Hoffmann T. C. Mulrow C. D. (2021). The PRISMA 2020 statement: an updated guideline for reporting systematic reviews. Bmj 372, n71. 10.1136/bmj.n71 33782057 PMC8005924

[B50] Rhee C. J. Sriram S. Ionchev A. Schreiber M. D. Meadow W. (2013). Acute haemodynamic effects of inhaled nitric oxide in premature infants with mild-to-moderate respiratory distress. Arch. Dis. Child. Fetal Neonatal Ed. 98, F183–F184. 10.1136/archdischild-2012-302308 22990132

[B51] Roberts J. D. Jr. Fineman J. R. Morin F. C. 3rd Shaul P. W. Rimar S. Schreiber M. D. (1997). Inhaled nitric oxide and persistent pulmonary hypertension of the newborn. The Inhaled Nitric Oxide Study Group. N. Engl. J. Med. 336, 605–610. 10.1056/NEJM199702273360902 9032045

[B52] Rose M. J. Stenger M. R. Joshi M. S. Welty S. E. Bauer J. A. Nelin L. D. (2010). Inhaled nitric oxide decreases leukocyte trafficking in the neonatal mouse lung during exposure to >95% oxygen. Pediatr. Res. 67, 244–249. 10.1203/PDR.0b013e3181ca0d93 19915514 PMC2829761

[B53] Schreiber M. D. Gin-Mestan K. Marks J. D. Huo D. Lee G. Srisuparp P. (2003). Inhaled nitric oxide in premature infants with the respiratory distress syndrome. N. Engl. J. Med. 349, 2099–2107. 10.1056/NEJMoa031154 14645637

[B54] Shea B. J. Reeves B. C. Wells G. Thuku M. Hamel C. Moran J. (2017). AMSTAR 2: a critical appraisal tool for systematic reviews that include randomised or non-randomised studies of healthcare interventions, or both. BMJ 358, j4008. 10.1136/bmj.j4008 28935701 PMC5833365

[B55] Speer C. P. (2006). Pulmonary inflammation and bronchopulmonary dysplasia. J. Perinatol. 26 (Suppl. 1), S57–S62. 10.1038/sj.jp.7211476 16625227

[B56] Srisuparp P. Heitschmidt M. Schreiber M. D. (2002). Inhaled nitric oxide therapy in premature infants with mild to moderate respiratory distress syndrome. J. Med. Assoc. Thai 85 (Suppl. 2), S469–S478. 12403222

[B57] Su P. H. Chen J. Y. (2008). Inhaled nitric oxide in the management of preterm infants with severe respiratory failure. J. Perinatol. 28, 112–116. 10.1038/sj.jp.7211881 17989696

[B58] Subhedar N. V. Ryan S. W. Shaw N. J. (1997). Open randomised controlled trial of inhaled nitric oxide and early dexamethasone in high risk preterm infants. Arch. Dis. Child. Fetal Neonatal Ed. 77, F185–F190. 10.1136/fn.77.3.f185 9462187 PMC1720712

[B59] ter Horst S. A. Walther F. J. Poorthuis B. J. Hiemstra P. S. Wagenaar G. T. (2007). Inhaled nitric oxide attenuates pulmonary inflammation and fibrin deposition and prolongs survival in neonatal hyperoxic lung injury. Am. J. Physiol. Lung Cell. Mol. Physiol. 293, L35–L44. 10.1152/ajplung.00381.2006 17384081

[B60] Thebaud B. Goss K. N. Laughon M. Whitsett J. A. Abman S. H. Steinhorn R. H. (2019). Bronchopulmonary dysplasia. Nat. Rev. Dis. Prim. 5, 78. 10.1038/s41572-019-0127-7 31727986 PMC6986462

[B61] Thom C. S. Devine M. Kleinman S. Jensen E. A. Lambert M. P. Padula M. A. (2020). Neonatal platelet count trends during inhaled nitric oxide therapy. Br. J. Haematol. 188, e28–e30. 10.1111/bjh.16301 31840227 PMC6982552

[B62] Van Meurs K. P. Hintz S. R. Ehrenkranz R. A. Lemons J. A. Ball M. B. Poole W. K. (2007). Inhaled nitric oxide in infants >1500 g and <34 weeks gestation with severe respiratory failure. J. Perinatol. 27, 347–352. 10.1038/sj.jp.7211690 17443204

[B63] Van Meurs K. P. Wright L. L. Ehrenkranz R. A. Lemons J. A. Ball M. B. Poole W. K. (2005). Inhaled nitric oxide for premature infants with severe respiratory failure. N. Engl. J. Med. 353, 13–22. 10.1056/NEJMoa043927 16000352

[B64] Vohr B. R. Wright L. L. Dusick A. M. Mele L. Verter J. Steichen J. J. (2000). Neurodevelopmental and functional outcomes of extremely low birth weight infants in the national institute of child health and human development neonatal research network, 1993-1994. Pediatrics 105, 1216–1226. 10.1542/peds.105.6.1216 10835060

[B65] Walsh M. C. Hibbs A. M. Martin C. R. Cnaan A. Keller R. L. Vittinghoff E. (2010). Two-year neurodevelopmental outcomes of ventilated preterm infants treated with inhaled nitric oxide. J. Pediatr. 156, 556–561.e1. 10.1016/j.jpeds.2009.10.011 20138299 PMC2843768

[B66] Wang Y. F. Liu C. Q. Gao X. R. Yang C. Y. Shan R. B. Zhuang D. Y. (2011). Effects of inhaled nitric oxide in neonatal hypoxemic respiratory failure from a multicenter controlled trial. Chin. Med. J. Engl. 124, 1156–1163. 21542988

[B67] Watson R. S. Clermont G. Kinsella J. P. Kong L. Arendt R. E. Cutter G. (2009). Clinical and economic effects of iNO in premature newborns with respiratory failure at 1 year. Pediatrics 124, 1333–1343. 10.1542/peds.2009-0114 19841128

[B68] Wei Q. F. Pan X. N. Li Y. Feng L. Yao L. P. Liu G. L. (2014). Efficacy of inhaled nitric oxide in premature infants with hypoxic respiratory failure. Zhongguo Dang Dai Er Ke Za Zhi 16, 805–809. 25140772

[B69] Wu F. Liu G. Feng Z. Tan X. Yang C. Ye X. (2019). Short-term outcomes of extremely preterm infants at discharge: a multicenter study from Guangdong province during 2008-2017. BMC Pediatr. 19, 405. 10.1186/s12887-019-1736-8 31685004 PMC6827215

[B70] Wu X. Feng Z. Kong J. Lai Y. Jia C. Xu Z. (2021). Efficacy and safety of surfactant administration via thin catheter in preterm infants with neonatal respiratory distress syndrome: a systematic review and meta-analysis. Pediatr. Pulmonol. 56, 3013–3025. 10.1002/ppul.25545 34215018

[B71] Zupancic J. A. Hibbs A. M. Palermo L. Truog W. E. Cnaan A. Black D. M. (2009). Economic evaluation of inhaled nitric oxide in preterm infants undergoing mechanical ventilation. Pediatrics 124, 1325–1332. 10.1542/peds.2008-3214 19841125

